# Evaluation of Optic Nerve Head Parameters, Retinal Nerve Fiber Layer Thickness, and Ocular Perfusion Pressure in Migraine Patients

**DOI:** 10.7759/cureus.4599

**Published:** 2019-05-04

**Authors:** Ngu Dau Bing Michael, Adil Hussein, Sanihah ABD Halim, Siti Azrin AB Hamid

**Affiliations:** 1 Ophthalmology, School of Medical Sciences, Universiti Sains Malaysia, Kota Bharu, MYS; 2 Ophthalmology, Hospital Universiti Sains Malaysia, Kota Bharu, MYS; 3 Neurology, School of Medical Sciences, Universiti Sains Malaysia, Kota Bharu, MYS; 4 Biostatistics and Research Methodology, School of Medical Sciences, Universiti Sains Malaysia, Kota Bharu, MYS

**Keywords:** migraine, retinal nerve fiber layer, optic nerve head, ocular perfusion pressure

## Abstract

Background

Neurovascular changes occur during the migraine is believed to cause alteration in cerebral and retinal circulation that possible result in damage to the brain and even retina or optic nerve. Retinal nerve fiber layer (RNFL) thickness measurement can be used as an index to assess ganglion cell and retinal nerve fiber damages. The aim of this study was to evaluate the optic nerve head (ONH) parameters, RNFL thickness, and ocular perfusion pressure (OPP) in migraine patients.

Methods

This was a cross-sectional study, conducted in Hospital Universiti Sains Malaysia, Kelantan from July 2016 to November 2018, involving patients with a confirmed diagnosis of migraine and controls. Ninety-four eyes of 47 migraine patients and 94 eyes of 47 healthy subjects were included in this study. Blood pressure and intraocular pressure were measured and OPP was calculated. ONH parameters and RNFL thickness were measured using optical coherence tomography (OCT) after pupillary dilatation. Statistical analysis was done using Statistical Package for the Social Science (SPSS Inc Version 24).

Results

With respect to all means values of ONH parameters, there was no statistically significant difference between migraine patients and controls. For RNFL, there were significant reductions in average and superior RNFL thickness on both eyes with adjustment of age and gender (*P*-value: right eye (RE) average = 0.027; RE superior = 0.034; left eye (LE) average = 0.037; LE superior = 0.031). In view of OPP, there was no significant difference between migraine patients and controls (*P*-value = 0.172). Weak correlations were found between the ONH parameters and RNFL thickness with OPP, respectively, in migraine patients.

Conclusion

This study showed no difference in ONH parameters between migraine patients and healthy subjects. There was significant thinning in average and superior RNFL for migraine patients. No difference found in OPP between both groups. ONH parameters and RNFL thickness had a weak correlation with OPP in migraine patients.

## Introduction

Migraine is a primary headache, often described as an episodic, recurring throbbing pain that may last from hours to 2-3 days. Based on the WHO reports, Atlas of Headache Disorders and Resources in the World 2011, migraine affects 11% of adults worldwide, and females have three times higher prevalence than males [1]. According to the International Classification of Headache Disorders, 3rd edition (ICHD-3) in 2013, two major groups of migraine are migraine without aura and migraine with aura [2].

The mechanism of migraine is complex and it was once believed to be caused by vasodilation, while aura was believed to result from vasoconstriction. The current state of knowledge suggests that neurovascular dysfunction leads to sequence changes in intracranial and extracranial, and each phase of migraine is probably mediated by different neuroanatomical structures [3]. This neurovascular changes will lead to vasospasm of cerebral and retrobulbar vessels. Although it is just a transient phenomenon, the chronicity of the disease is believed to be a risk factor for structural damage to the brain and even retina or optic nerve due to the reduction in blood flow of optic nerve head (ONH) [4]. The perfusion quality alterations in ONH microcirculation may contribute to ganglion cell death in migraine patients [5]. Hence, ONH parameters and retinal nerve fiber layer (RNFL) thickness are likely to be affected by the disease based on the neurovascular mechanism.

Using optical coherence tomography (OCT), the quantitative measurement of ONH parameters and RNFL thickness will distinguish a normal from abnormal optic disc and RNFL. Moreover, OCT is a non-invasive and non-contact imaging technique that can be easily performed in the clinic. The aim of this study was to compare the ONH parameters, RNFL thickness, and ocular perfusion pressure (OPP) between migraine patients and healthy subjects.

## Materials and methods

This was a cross-sectional study conducted in Hospital Universiti Sains Malaysia, Kelantan from July 2016 to November 2018. This study was conducted in accordance with the declaration of Helsinki and was approved by local ethical boards [USM/JEPeM/16090341].

The sample size was calculated using G Power 3.1.9. Forty-seven migraine patients and 47 age-matched healthy controls were included in this study. Patients with migraine were recruited from the neurology clinic and the control group was recruited from the ophthalmology clinic. No sampling method applied for migraine patients; meanwhile, a simple random sampling applied for controls. Inclusion criteria for migraine were adult patient (above 18 years old) who had confirmed the diagnosis by a neurologist based on the ICHD-3 criteria for migraine with aura and migraine without aura. The inclusion criteria for healthy controls were the same age group with the absence of any pathological headache. Excluded were subjects with known systemic diseases (such as diabetes mellitus and hypertension) or known ophthalmic disorders, refractive error exceeding ± 3.0 diopters and had previously undergone any type of eye surgery. Patients with a history of central nervous system disorders including brain tumors, infarction, epilepsy, Alzheimer’s disease, and any type of headache except for migraine were excluded from this study. Subjects with optic disc anomaly and any retinal disease were also excluded from the study. Written informed consent was obtained from all the participants.

The demographic data (patients’ age, gender, and ethnicity) was obtained from either the patients or their medical record. Information such as the duration of disease and frequency of attack was obtained directly from the patients. All the patients that fulfilled the inclusion criteria underwent a complete both ocular examination including slit-lamp examination, dilated fundus examination, measurement of intraocular pressures (IOP) and blood pressure (BP) of both eyes. This was followed by the measurement of the ONH parameters and RNFL thickness of both eyes using Cirrus HD-OCT 4000 (Carl-Zeiss Meditec Inc., USA), which performs low coherence interferometry with an 840-nm superluminescent light-emitting diode to produce high-resolution tomograms. Data for ONH parameters and RNFL were acquired using the ‘Optic Disc Cube 200x200’ protocol, after pupil dilatation. For the measurement of RNFL thickness, a 3.46-mm diameter centered on the optic disc was measured. All four quadrants were included for analysis in this study; superior, inferior, temporal and nasal. Only a well-focused scan with a signal strength of greater than seven was included. OCT findings of both eyes were taken for analysis to determine any laterality effect by disease.

Both eyes IOP were measured using Reichert 7CR (Ametek Inc, Pennsylvania, United States), which is a non-contact tonometer prior to pupillary dilatation. BP was measured by a digital blood pressure monitor (Omron) twice, five minutes apart. Due to diurnal variation, the examination of a patient with BP and IOP was taken between 10 am to 12 pm. OPP was derived from the equation, 2/3[diastolic BP +1/3(systolic BP-diastolic BP)] - IOP. The average of IOP values from both eyes were used for OPP calculation.

The statistical analysis was carried out using the Statistical Package for Social Sciences (SPSS) Version 24. Analysis of covariance (ANCOVA) was applied to compare the ONH parameters, RNFL thickness, and OPP between migraine patients and controls with adjustment of age and gender. The significant difference value was determined by *P*-value <0.05. The correlation between the ONH parameters and RNFL with OPP among migraine patients was tested using Pearson correlation. The correlation coefficient, *r,* less than 0.3 indicates a weak correlation, between 0.3 and 0.7 consider moderate correlation, and larger than 0.7 would suggest a strong correlation. The sign of *r* indicates the direction of the linear relationship.

## Results

The distribution of demographic data is shown in Table 1. A total of 94 subjects (47 migraine patients and 47 age and gender-matched controls) were recruited in this study. The 94 subjects included 16 males (17.0%) and 78 females (83.0%) with a mean age of 41.5 ± 12.36 years, ranging from 19 to 71 years old. Among all subjects, 81 are Malays (86.2%), followed by 11 Chinese (11.7%) and two Indians (2.1%).

**Table 1:** Baseline demographic characteristics ^a^Fisher exact test was applied; ^b^independent *t-*test was applied, significant level set at 0.05. SD, standard deviation; SBP, systolic blood pressure; DBP, diastolic blood pressure; RE IOP, right eye intraocular pressure; LE IOP, left eye intraocular pressure; OPP, ocular perfusion pressure

Variables	All (n = 94)	Migraine (n = 47)	Controls (n = 47)	p-value
Age , Mean (SD)	41.5 (12.36)	41.5 (12.54)	41.6 (12.32)	0.967^b^
Gender				
Male, n (%)	16 (17.0)	8 (17.0)	8 (17.0)	0.608^a^
Female, n (%)	78 (83.0)	39 (83.0)	39 (83.0)	
Ethnicity				
Malay, n (%)	81 (86.2)	41 (87.2)	40 (85.1)	0.950^a^
Chinese, n (%)	11 (11.7)	5 (10.6)	6 (12.8)	
Indian, n (%)	2 (2.1)	1 (2.1)	1 (2.1)	
SBP (mmHg), Mean (SD)	126.6 (12.54)	127.5 (12.86)	125.7 (12.29)	0.493^b^
DBP (mmHg), Mean (SD)	78.8 (8.18)	80.6 (7.24)	76.9 (8.69)	0.025^b^
RE IOP (mmHg), Mean (SD)	13.5 (2.37)	13.7 (2.65)	13.3 (2.07)	0.516^b^
LE IOP (mmHg), Mean (SD)	13.5 (2.65)	13.8 (3.04)	13.2 (2.18)	0.261^b^
OPP (mmHg), Mean (SD)	49.7 (5.75)	50.5 (5.31)	48.9 (6.11)	0.179^b^
Duration of migraine (years)				
Less than 15, n (%)		28 (59.6)		
15 and above, n (%)		19 (40.4)		
Frequency of attack (times/per month)				
Less than 5, n (%)		32 (68.1)		
5 and above, n (%)		15 (31.9)		

Nineteen migraine patients (40.4%) had suffered the disease for 15 years or more, while 28 of them (59.6%) had migraine less than 15 years. Thirty-two patients (68.1%) had a frequency of migraine attack less than five times per month. Both groups’ mean BP and IOP were within the normal range. The diastolic BP in the migraine group (80.6 mmHg, SD 7.24) was significantly higher than the control group (76.9 mmHg, SD 8.69; *P*-value = 0.025) although both groups were normotensive.

With respect to all mean values of ONH parameters, there were no statistically significant differences between migraine patients and controls with adjustment of age and gender (Table 2). Meanwhile, the RNFL thickness in the average and superior quadrants of both eyes were found significantly lower in migraine patients, as compared to the healthy subjects (*P*-value: RE average = 0.027; RE superior = 0.034; LE average = 0.037; LE superior = 0.031; Table 3).

**Table 2:** Comparison of the ONH parameters on the right eye and left eye between migraine patients and controls with adjustment of age and gender (n = 94) ^a^ANCOVA was applied, significant level set at 0.05. ONH, optic nerve head; SD, standard deviation; CI, confidence interval, CDR, cup-to-disc-ratio; ; ANCOVA, analysis of covariance

ONH Parameters	Migraine	Mean (SD)	Mean diff. (95% CI)	P-value^a^
Right Eye				
Disk area (mm^2^)	Yes	2.15 (0.39)	0.01 (-0.14, 0.17)	0.869
	No	2.16 (0.36)		
Rim area (mm^2^)	Yes	1.34 (0.26)	0.04 (-0.06, 0.14)	0.452
	No	1.38 (0.23)		
Cup volume (mm^3^)	Yes	0.22 (0.14)	-0.01 (-0.07, 0.07)	0.956
	No	0.21 (0.21)		
Average CDR	Yes	0.58 (0.11)	-0.02 (-0.07, 0.03)	0.484
	No	0.57 (0.12)		
Vertical CDR	Yes	0.54 (0.12)	-0.03 (-0.08, 0.02)	0.256
	No	0.51 (0.11)		
Left Eye				
Disk area (mm^2^)	Yes	2.14 (0.32)	0.04 (-0.09, 0.18)	0.524
	No	2.18 (0.34)		
Rim area (mm^2^)	Yes	1.32 (0.23)	0.04 (-0.06,0.13)	0.443
	No	1.36 (0.21)		
Cup volume (mm^3^)	Yes	0.22 (0.15)	0.00 (-0.07, 0.07)	0.913
	No	0.22 (0.18)		
Average CDR	Yes	0.59 (0.12)	-0.01 (-0.06, 0.04)	0.676
	No	0.58 (0.12)		
Vertical CDR	Yes	0.55 (0.12)	-0.02 (-0.06, 0.03)	0.590
	No	0.53 (0.11)		

**Table 3:** Comparison of the mean RNFL on right eye and left eye between migraine patients and controls with adjustment of age and gender (n = 94) ^a^ANCOVA was applied, significant level set at 0.05. RNFL, retinal nerve fiber layer; SD, standard deviation; CI, confidence interval; ANCOVA, analysis of covariance

RNFL	Migraine	Mean (SD)	Mean diff. (95% CI)	P-value^a^
Right Eye				
Average	Yes	95.23 (6.36)	3.09 (0.37, 5.81)	0.027
	No	98.30 (7.90)		
Superior	Yes	115.89 (13.41)	5.97 (0.35, 11.59)	0.034
	No	121.83 (14.72)		
Temporal	Yes	69.21 (11.00)	1.90 (-2.19, 5.95)	0.362
	No	71.11 (9.03)		
Inferior	Yes	125.38 (11.45)	3.89 (-1.10, 8.87)	0.132
	No	129.23 (14.25)		
Nasal	Yes	70.38 (9.50)	0.14 (-3.87, 4.15)	0.944
	No	70.51 (10.16)		
Left Eye				
Average	Yes	95.15 (5.31)	2.81 (0.23, 5.39)	0.037
	No	97.94 (8.02)		
Superior	Yes	122.83 (9.85)	5.75 (0.64, 10.87)	0.031
	No	128.55 (15.72)		
Temporal	Yes	67.17 (9.70)	3.41 (-0.61, 7.42)	0.099
	No	70.55 (10.65)		
Inferior	Yes	123.11 (11.86)	2.05 (-2.50, 6.60)	0.380
	No	125.13 (11.28)		
Nasal	Yes	67.43 (8.22)	-0.49 (-4.09, 3.13)	0.790
	No	66.94 (9.22)		

In view of OPP, no statistically significant difference was found between the migraine group and control group (*P*-value = 0.172; Table 4). However, migraine patients had a higher mean value of OPP (50.46 mmHg) than controls (48.85 mmHg).

**Table 4:** Comparison of the mean OPP between migraine patients and controls with adjustment of age and gender (n = 94) ^a^ANCOVA was applied, significant level set at 0.05. OPP, ocular perfusion pressure; SD, standard deviation; CI, confidence interval; ANCOVA, analysis of covariance

Migraine	Mean (SD)	Mean difference (95% CI)	P-value^a^
Yes	50.46 (5.31)	-1.61 (-3.93, 0.71)	0.172
No	48.85 (6.11)		

The result for the correlation between ONH parameters and OPP in migraine patients was reported in Table 5. All ONH parameters showed a weak negative correlation with OPP, except for the right eye disc area and both eyes rim area showed a weak positive correlation (all *r* < 0.3). With regard to RNFL thickness, all four quadrants of RNFL showed a weak positive correlation with OPP, except for the superior quadrant of the left eye which is negatively correlated with OPP in migraine patients (*r* < 0.3; Table 6).

**Table 5:** Correlation of the mean ONH parameters and mean OPP in migraine patients (n = 47) Pearson’s product-moment correlation was used. ONH, optic nerve head; OPP, ocular perfusion pressure; CDR, cup-to-disc-ratio

ONH Parameters	Correlation Coefficient, r (p-value)	
	Right Eye	Left Eye
Disc area (mm^2^)	0.05 (0.735)	-0.16(0.277)
Rim area (mm^2^)	0.21 (0.153)	0.04(0.781)
Cup volume (mm^3^)	-0.21 (0.163)	-0.13(0.388)
Average CDR	-0.21 (0.153)	-0.18(0.219)
Vertical CDR	-0.24 (0.104)	-0.21(0.160)

**Table 6:** Correlation of the mean RNFL thickness and mean OPP in migraine patients (n = 47) Pearson’s product-moment correlation was used. RNFL, retinal nerve fiber layer; OPP, ocular perfusion pressure

RNFL Thickness	Correlation Coefficient, r (p-value)	
	Right Eye	Left Eye
Average	0.26 (0.081)	0.09 (0.568)
Superior	0.22 (0.134)	-0.11 (0.444)
Temporal	0.20 (0.190)	0.07 (0.620)
Inferior	0.04 (0.773)	0.13 (0.381)
Nasal	0.09 (0.540)	0.07 (0.632)

## Discussion

Migraine is a chronic, progressive neurovascular disorder and can cause several complications, including retina ischemia secondary to central artery occlusion [6-7]. This is possible due to higher resistance in the central retinal artery and posterior ciliary artery during a migraine attack or headache-free period [8]. The main source of blood supply to the ONH is the posterior ciliary artery circulation, except for the superficial nerve fiber layer which is supplied mainly by the central retinal artery [9]. It seems reasonable that an alteration in blood supply to the ONH will lead to ganglion cell death [10].

The mean age of migraine patients in this study was 41.5 years (range: 19 to 71 years). This was fairly consistent with several other studies [11-12]. Our study reported a female preponderance of disease which was similar to the WHO reports [1]. The majority of the participants in our study were Malays. This is because Kelantan is a predominantly Malay village in the north-eastern state of Peninsular Malaysia.

We examined the ONH parameters in migraine patients and did not find any significant differences between study and control groups. This finding was consistent with other authors’ studies [5,13]. This could be possible due to the lack of sensitivity of the OCT machine in detecting the subtle optic disc changes in migraine patients. Furthermore, no significant difference in IOP was detected between migraine patients and healthy group. Moehnke et al. used a different instrument, confocal scanning laser ophthalmoscope, for ONH topographical analysis and also found that there was no difference between migraine patients and age-matched control subjects [14]. A single measurement just provides parameter information, but the true value of repeated measurement may help provide information about the potential influence of migraine to the ONH.

Based on the literature review, the results of RNFL thickness in migraine have not been very consistent. Some authors observed that the mean RNFL was thinner, while others reported only a thinner RNFL in a specific individual quadrant [11,15-18]. These distinct results may be due to the use of different methods and sample sizes, racial differences, and lack of standardized in terms of including and excluding criteria for the subjects. One of the earliest studies about the measurement of RNFL thickness in migraine patients by Tan et al. in 2005 had concluded that RNFL thickness is unaffected by the disease [19]. However, in our study, we found that there was a statistically significant reduction of average and superior RNFL thickness in migraine patients compared to healthy controls. The discrepancies between our results and Tan et al.’s could be due to different evaluative techniques whereby they used scanning laser polarimetry for measurement. In addition, relatively small sample size was also their limitation. On another hand, our findings were consistent with other studies [15-16]. This could be due to the similar OCT machines, which was Cirrus HD-OCT (Carl Zeiss) used in the measurement of RNFL thickness. Selective superior RNFL thinning in our study might be explained by the vulnerability of retinal axons to ischemia. This result supports the hypothesis that migraine can result in a decrease in cerebral blood flow and even retinal circulation.

We found no difference in OPP between migraine patients and control group. This result is possible as most of the migraine patients were in the attack-free period during the examination. Furthermore, OPP was measured during daytime which could be different at night as IOP and BP fluctuate throughout 24 hours. However, we noted that diastolic BP in migraine patients was statistically significantly higher than the controls. Similar findings were reported in previous studies [20-22]. The mechanism suggested was the renin-angiotensin system that involved in hypertension has activities in the central nervous system that may be related to migraine pathogenesis [23]. Furthermore, angiotensin-converting enzyme inhibitors are effective for migraine treatment [24].

There were poor correlations between ONH parameters or RNFL thickness and OPP in migraine patients. To our knowledge, this was the first study to study the correlation of ONH parameters and RNFL thickness with OPP in migraine patients. Our result showed that OPP is not a risk factor for ONH and RNFL changes in migraine patients. However, this needs further investigation such as measuring BP and IOP over 24 hours which will provide a better value of OPP. This correlation was different in glaucoma patients, whereby OPP was positively associated with RNFL thickness [25]. In fact, studies had been proven that OPP is associated with prevalence or progression of glaucoma [26-27]. This is due to the abnormal autoregulation of ocular blood flow in glaucoma patients [28]. Lately, newer technology has been used to assess the ONH perfusion via OCT-based microangiography [29]. This technique could help us visualize and quantify ONH perfusion that will provide a better picture of vasculopathy at ONH.

This study has several limitations. First, we did not assess ONH parameters and RNFL thickness based on the severity of the disease. The migraine disability assessment score (MIDAS) is a well-known tool to assess the severity of migraine. Another limitation of the study is no assessment of visual function such as color vision, visual field and visual contrast in migraine patients. Anatomically there was a reduction in RNFL thickness in this study; however, we did not assess the functionality of the visual system in relation to anatomical finding. Thirdly, the limitation of this study is the instruments used for BP and IOP measurement. Manual blood pressure measurement, instead of a digital sphygmomanometer, will provide better accuracy of the readings. The same is applied to IOP measurement. Goldmann applanation tonometry will result in more accurate IOP reading than air puff tonometry.

## Conclusions

In conclusion, ONH parameters were not affected in migraine patients. There was significant thinning in the average and superior RNFL for migraine patients. No difference in OPP in migraine patients compared with healthy subjects. Weak correlations were found between ONH parameters or RNFL thickness with OPP in migraine patients.
